# Effective Treatment of Chronic Cough with Tinidazole as the Newest Antiprotozoa against *Lophomonas blattarum*

**DOI:** 10.1155/2022/2413941

**Published:** 2022-11-03

**Authors:** Zahra Anhaee Nasseri, Majid Mirsadraee, Mahsa Manafi Varkiani, Younes Ghaderi, Fariba Berenji, Shadi Ghaffari

**Affiliations:** ^1^Department of Internal Medicine, Faculty of Medicine, Islamic Azad University, Mashhad Branch, Mashhad, Iran; ^2^Clinical Professor, Internist and Pulmonologist, Department of Internal Medicine, Medical School of Islamic Azad University, Mashhad Medical Sciences Branch, Mashhad, Iran; ^3^Resident in Cardiology, Department of Cardiology, Imam Reza Hospital, Mashhad University of Medical Sciences, Mashhad, Iran; ^4^Department of Parasitology, Mashhad University of Medical Sciences, Mashhad, Iran; ^5^Department of Physiology, Faculty of Biology, Islamic Azad University, Damghan, Iran

## Abstract

**Background:**

Chronic cough is a common problem in the setting of family physicians. Recently, *Lophomonas blattarum* was considered a cause of respiratory symptoms in children and adults.

**Objective:**

This study is aimed at determining the effect of antiprotozoal treatment of *Lophomonas* in patients with a chronic cough in Mashhad during 2020-2021.

**Materials and Methods:**

This study was a randomized clinical trial. In this study, 60 patients with chronic cough and unremarkable imaging findings, who were unresponsive to three steps of standard treatment, were randomly assigned to the treatment, with 2 weeks of tinidazole and placebo. The tinidazole and placebo were prepared in a completely identical shape, and a random assignment was performed by a third party. The primary outcome was a complete resolution of cough. A follow-up of treatment was performed. Data were analyzed using the SPSS software version 25.

**Results:**

The basic demographic results showed no significant differences of sex and age between two groups. The results of this study showed a complete resolution of all respiratory symptoms in 40% (12), a complete improvement of cough in 40% (12), and a complete resolution of dyspnea in 50% (10) of the tinidazole group. The remaining showed significant improvement in the severity of cough and dyspnea. Postnasal drip, sputum, body temperature, and airway hyperresponsiveness were improved significantly. After tinidazole treatment, laboratory assessment of bronchial lavage and sputum revealed that 86 percent of smears were converted to negative.

**Conclusion:**

Tinidazole effectively resolved the chronic cough and most of the respiratory symptoms. *Lophomonas blattarum* is a potential mechanism for chronic cough.

## 1. Introduction

Cough is a protective reflex for the airways and lungs. However, persistent cough is considered a respiratory problem which requires management. The main cause of cough in 78-99% of patients can be identified in the clinic, and its treatment is effective in 68 to 99% of patients. However, despite diagnostic procedures, the cause of 47 to 46% of patients with chronic cough remains unclear [1]. Therefore, new etiological causes may exist for subjects who suffer from resistant cough and normal chest roentgenogram, which do not fulfill the diagnostic criteria of asthma, gastroesophageal reflux disease, and chronic rhinosinusitis.

**Figure 1:**
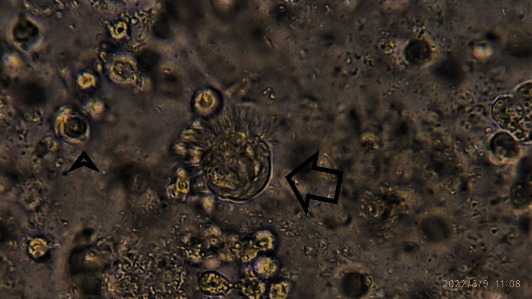
*Lophomonas blattarum* in the sputum sample, note to flagellate in half of the surface of the protozoa (large arrow represents *Lophomonas*; arrowhead represents neutrophil).

*Lophomonas* is a flagellate protozoon that lives in the intestines of insects and beetles. *Lophomonas striata* (*L. striata*) and *L. blattarum* are two species of *Lophomonas* [2]. *L. blattarum* structural was distinguished by a light microscope in 1911 and later showed to influence on human disease especially lung disease. The shape of *L. blattarum* is usually round, oval or piriform, ranging in size from 15 to 40 micrometers, as well as a many flagella extending from the anterior end of the organism, and the phagocytic vacuoles can be found in the cytoplasm [3] (Figure 1).

This protozoa was firstly considered a rare cause of bronchopulmonary inflammation as well as respiratory symptoms [4]. Recent studies showed the increasing frequency of this organism in adults, with bronchopulmonary disease and asthma, as well as children with severe lung disease [5]. The clinical symptoms of people with *Lophomonas* are similar to those of other respiratory illnesses such as asthma, pneumonia, and bronchiectasis [6]. Fever was observed in 90% of patients in the range from 37.5 to 39°C [6]. As a diagnostic problem, it is difficult to differentiate *Lophomonas blattarum* from epithelial cells under a light microscope; therefore, many experts neglect its role in respiratory diseases [3]. Recent study, in our local region, on the samples of bronchial lavage and nasal secretion, showed 45 subjects out of 133 candidates were positive for *Lophomonas* infection [7]. Five of six samples, produced from nasal secretion smears, were positive for *Lophomonas* [7] in this investigation. This investigation revealed that delaying in the identification and treatment of *Lophomonas* increases the likelihood of developing problems such as a protracted and annoying cough [7].

We believe that the *Lophomonas blattarum* has a major role in the etiology of cough in our region (east of the Iran), and in terms of the low impact of experimental anti-*Lophomonas* treatment and some side effects reported from metronidazole, further investigations about the best treatment of subjects who suffer from cough due to *Lophomonas blattarum* are required. This study is aimed at determining the effect of tinidazole (as the most developed anti-*Lophomonas* treatment) in the patients with difficulty in treating chronic cough.

## 2. Materials and Methods

This study was performed as a double-blinded, phase 3, randomized clinical trial on the efficacy of tinidazole treatment against *Lophomonas blattarum* in the subjects suffering from chronic cough. The article was accepted by the Ethics Committee of the Central Organization of Islamic Azad University of Mashhad (IR.IAU.MSHD.REC.1399.088) and in the Iranian Registry of Clinical Trials https://www.irct.ir/ (IRCT20091111002695N10). All patients with chronic cough were visited in a private Lung Clinic in Mashhad, Northeast Iran, from the 5th of Dec 2020 until the 5th of March 2021 were entered to this study. Clinical investigations were directed toward identifying asthma, gastroesophageal reflux, and persistent atypical infections such as rhinosinusitis. As a pretrial phase, all subjects were treated with inhaled corticosteroids for one month, doxycycline for 15 days, and/or proton pump inhibitors for one month, following a previously described stepwise method [8]. Subjects who were resistant to the initial treatment were included in this study, irrespective of result of sputum analysis for parasite. Exclusion criteria included abnormal initial chest x-ray, intolerance to oral medications, chronic and proven asthma, tuberculosis, GERD, ear wax, COPD, lung cancer, and consuming angiotensin-converting enzyme inhibitors.

**Figure 2:**
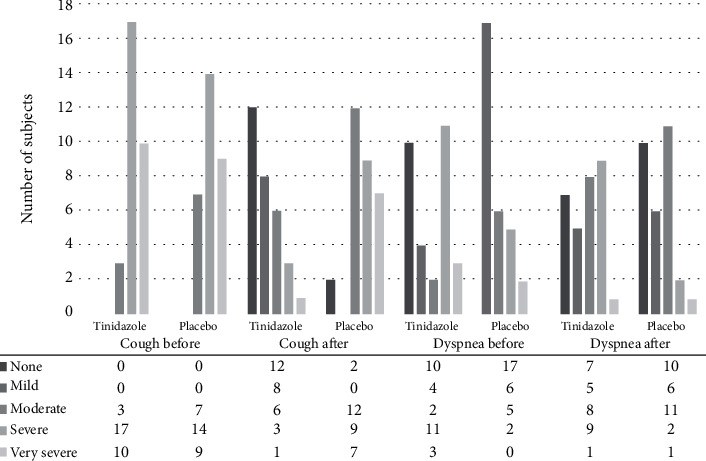
Frequency of cough and dyspnea as graded by clinical staging by the authors, before and after treatment with tinidazole and placebo in subjects suffering from persistent cough.

The study included bronchial lavage fluid (BLF) as a standard diagnostic procedure for all subjects, but a sputum sample was taken if the patients did not accept bronchoscopy. Samples were divided into two parts, one for *Lophomonas* and the other for cytology in which the classification of sputum inflammatory cells was determined. Samples were sent to the laboratory to a tertiary university hospital (Imam Reza Hospital, Mashhad University of Medical Sciences, Iran) for evaluation of the *Lophomonas* by an experienced technician. The smears were stained in trichrome and examined under a light microscope (Figure 2). The primary outcome was the improvement of cough by an antiprotozoal agent (tinidazole). For a better understanding of the evolution of respiratory symptoms, we used a staging from mild to severe form.

The trial was started after the sampling, and therefore, regardless of the positive or negative result of the sputum sample or bronchial lavage, antiprotozoal treatment or placebo was started. Random assignment of patients to two groups of the drug (tinidazole) and placebo was performed using a table of random numbers and double blocks. Assignment was performed blindly by a staff in the pharmacy, and he offered the drugs to the subjects sequentially according to the numbers included in the code list. Tinidazole tablets containing 500 mg of Pars Daru Company were administered every 12 hours for 15 days to participants in the experimental group. Vitamin B_1_ at a dosage of 300 mg from Galen Pharmaceutical Company was utilized as a placebo in the control group (placebo), since it was identical in form and size to the tinidazole. Tinidazole and the placebo repacked in the identical boxes were undetectable by the physician and the patient. Dextromethorphane as needed was added to both groups, in case of annoying cough. After 15 days, at the final stage, the clinical findings such as dyspnea and cough, the staging of these symptoms, and drug side effects were evaluated. At the final stage, bronchoscopy was not performed (as ethical issue due to invasiveness of this procedure). Therefore, sputum analysis obtained by an induced sputum by 5% sodium chloride via a nebulizer was replaced to bronchoscopy. We considered the response to the experimental treatment as a confirmation of the clinical diagnosis of *Lophomonas*, although we considered that some resistant *Lophomonas* may remained. A direct communication channel (telephone number) was provided between the patient and the executor of the project, and the patient was contacted once a week, in which the patient was asked about the side effects of the drug, and as soon as we became aware of any complication, the drug was discontinued.

### 2.1. Statistical Analysis

Sample size (sixty subjects divided to two groups) was calculated according to the low frequency of disease, 0.05 error, and 80% potency. After ensuring that the data were normal, relevant statistical tests such as the Student's *t*-test, paired *t*-test, analysis of variance, Mann–Whitney, Kruskal-Wallis, and chi-square were employed to compare two groups and evaluate changes after the experiment. The software used in this research was the IBM-SPSS version 25. Significance level of the tests is less than 5% (*p* < 0.05).

## 3. Results

Basic demographic results showed no significant differences of sex and age between two groups (Table 1). The mean temperature in the intervention and placebo groups before the intervention was 37.2 ± 0.26°C and 37.28 ± 0.22°C, and after the intervention were 36.99 ± 0.20°C and 37.16 ± 0.13°C, respectively, which showed a significant decrease in both groups. Cough as the primary outcome showed that all subjects in the tinidazole group suffered from moderate to very severe cough before the intervention. After the trial, 42% of them completely improved, and 60% showed significant improvement in cough severity score (Table 2, Figure 2). Postnasal drip, airway hypersensitivity, sputum, and shortness of breath (both frequency and severity) were significantly reduced in the tinidazole group (Table 2). Shortness of breath was reported in two-thirds of subjects, which in the tinidazole group were completely improved in 17 (56%) and significantly decreased in 43% of subjects (13 subjects) (Table 2, Figure 2). Sputum and bronchial lavage cytology revealed no significant difference in inflammatory cells obtained by induced sputum or bronchial lavage smear (Table 2). Overall results of treatment showed complete resolution of all respiratory symptoms in 12 (40%) subjects of tinidazole and 2 (6.7%) subjects of the placebo group (*p* = 0.001). Significant sense of well-being was revealed in 12 (40%) of the tinidazole and 4 (13%) of the placebo group (*p* = 0.001). Before the intervention, *Lophomonas* was observed in 53.5% of the patients, in the sputum of the tinidazole group, and after the intervention, the positive *Lophomonas* sputum smear was 13.3%, which showed a significant decrease (Table 2). Side effects of the tinidazole included nausea, vomiting, headache, melanuria, itching, constipation, and metallic taste in two subjects which was not significantly more than the placebo group. Long-term follow-up in the tinidazole group for one year showed a relapse of symptoms especially cough in 11 (18%) subjects.

## 4. Discussion

In the present study, the tinidazole was used for the treatment of the subjects who suffered from severe chronic cough who were not responsive to the three-step treatment of asthma, GERD, and chronic rhinosinusitis. The study's findings indicated a substantial decrease in the intensity of cough, shortness of breath, airway hypersensitivity, sputum, and PND. These findings were consistent with a reduction in the quantity of *Lophomonas* in sputum or bronchial lavage samples after the tinidazole therapy (lowered from 53.5 percent before the intervention to 13.3 percent after the intervention in the tinidazole group). However, in the control group, these changes in the symptoms and the amount of *Lophomonas* were not significant, which means that the *Lophomonas* parasite should be considered a potential cause of cough and other respiratory symptoms. We experienced significant improvement in many subjects who suffered from very severe cough which had not responded to all cough suppressants and led to disruption of daily activity and sleep.

Ribas et al. [9] reported the treatment of *Lophomonas* by metronidazole to reduce the protozoan (in sputum samples) and respiratory symptoms in immunocompromised patients. Shi et al. [10] conducted a study on the patients with bacterial pneumonia, who had upper respiratory tract symptoms, such as cough and shortness of breath. They found this parasite in sputum samples, and they reported that the treatment with oral metronidazole significantly reduced the symptoms and the number of parasites, in sputum smear or bronchoalveolar lavage. This study is similar in the present study, but our subjects were more selective.

Most of the subjects suffering from cough have been managed by treatment of asthma, gastroesophageal reflux, and postnasal drip. But we should keep in mind that these subjects suffering from *Lophomonas blattarum* are resistant to these treatments, and identification of this parasite and curative eradication of *Lophomonas* may lead to successful discontinuation of cough, which may not present in relapsing disease such as asthma and gastroesophageal reflux. This high gain has a cost. Its cost is performing bronchoscopy, which is an invasive and expensive procedure. Therefore, two important issues will be introduced here: (1) the diagnosis of *Lophomonas* in the present study and other studies was wet preparation of sputum and bronchial lavage fluid samples, which showed significant differences after the treatment with tinidazole. Therefore, wet preparation of bronchial lavage should be considered, in lung specialist clinics, in case of performing bronchoscopy in subjects suffering from resistant cough. (2) As bronchoscopy is not available in the setting of primary health care, we need new replacing accurate diagnostic methods to identify the *Lophomonas* easily in the specimens such as sputum. The main target of this attempt is to reduce the risk of missing subjects suffering from infection with *Lophomonas*, leading to the resistant cough.

In our experience, the recurrence of the cough in these patients is common (unpublished data). In this context, we found that the inflammatory response and the consequent inflammatory cell reactions in the sputum smear were not significant. We believe that this protozoa is noninvasive in the mucosal membrane of the respiratory tract, and therefore, immune response is not strong, to prevent the recurrences of this disease. Therefore, we should emphasize that, for the time being, our management of this disease is solely the use of available antiprotozoal drugs, such as metronidazole and tinidazole, and defense mechanisms leading by the immune system such as vaccination are not effective. The duration of tinidazole usage was lower according to classical references, but we believe that the excretion of tinidazole in the bronchial mucosa is not enough to kill the *Lophomonas* rapidly. Therefore, we used longer regimen, which was used in our region with more success, albeit with more side effects.

In conclusion, it should be noted that *Lophomonas* cough might be common in the worldwide and should be searched extensively in other geographical parts of the world. We recommend considering *Lophomonas* in subjects suffering from severe long-lasting resistant cough. The evaluation of this parasite is wet smear, which is cheap and widely available. Then, after confirming the diagnosis, treatment with imidazole drugs should be considered, but the side effects are the potential concern that physician and patients should be kept in mind. We recommend repeating the drug therapy without lab evaluation, if the symptoms recur after a couple of months.

## Figures and Tables

**Table 1:** Characteristics of demographic data of studied population (*n* = 60).

Variable	Categories	Tinidazole *n* = 30	Placebo *n* = 30	*p* value
Age (years), mean ± SD		52.86 ± 11.16	47.00 ± 13.01	0.060
Sex, *n* (%)	Male	21 (70.0%)	14 (46.7%)	0.067
	Female	9 (53.3%)	16 (53.3%)	

**Table 2:** Effect of tinidazole in course of treatment of *Lophomonas Blattarum* in subjects suffering from persistent cough.

Clinical findings	Score	Total	Before		After		*p* value
			Tinidazole	Placebo	Tinidazole	Placebo	
Temperature (°C)		37.2 ± 0.2	37.2 ± 0.26	37.2 ± 0.22	36.99 ± 0.20	36.99 ± 0.20	0.001^∗∗∗^
Cough	—	60 (100%)	30 (100%)	30 (100%)	18 (60%)	28 (94%)	0.001^∗^
Cough severity	No	0 (0%)	0 (0.0%)	0 (0.0%)	12 (40.0%)	2 (6.7%)	0.001^∗∗^
	Mild	0 (0%)	0 (0.0%)	0 (0.0%)	8 (26.7%)	0 (0%)	
	Moderate	10 (16%)	3 (10.0%)	7 (23.3%)	6 (20.0%)	12 (40%)	
	Severe	31(52%)	17 (56.6%)	14 (46.6%)	3 (10.0%)	9 (30%)	
	Very severe	19 (31%)	10 (33.3%)	9 (30%)	1 (3.3%)	7 (23.3%)	
PND	—	24 (40%)	12 (40.0%)	12 (40.0%)	4 (13.3%)	9 (30%)	0.021^∗^
Dyspnea	—	43 (72%)	20 (66.7%)	23 (76.6%)	13 (43.3%)	20 (66.7%)	0.016^∗^
Dyspnea severity	No	17 (28%)	10 (33.3%)	7 (23.3%)	17 (56.7%)	10 (33.3%)	0.001^∗∗^
	Mild	9 (15%)	4 (13.3%)	5 (16.7%)	6 (20.0%)	6 (20.0%)	
	Moderate	10 (17%)	2 (6.7%)	8 (26.7%)	5 (16.7%)	11 (36.7%)	
	Severe	20 (24%)	11 (36.7%)	9 (30%)	2 (6.7%)	2 (6.7%)	
	Very severe	4 (7%)	3 (10.0%)	1 (3.3%)	0 (0.0%)	1(3.3%)	
Sputum		38 (63%)	17 (56.7%)	21 (70%)	11 (36.7%)	18 (60%)	0.001^∗^
Airway sensitivity	—	39 (65%)	23 (67.7%)	16 (53.3%)	9 (30.0%)	14 (46.7%)	0.001^∗^
*Lophomonas* in sputum or BLF	—	25 (42%)	16 (53.3%)	9 (30%)	4 (13.3%)	5 (16.7%)	0.001^∗^
Inflammatory cells	Eosinophil	17 (28%)	9 (30.0%)	8 (26.7%)	8 (26.7%)	9 (30%)	0.564^∗∗∗∗^
	Neutrophil	23 (38%)	10 (33.3%)	13 (43.3%)	12 (40.0%)	12 (40%)	
	Mixed	7 (12%)	2 (6.7%)	5 (16.7%)	0 (0.0%)	3 (5%)	
	Paucigranulocytic	13 (22%)	9 (30.0%)	4 (13.3%)	10 (33.3%)	16 (26.7)	
Overall recovery	Complete improvement				12 (40%)	2 (6.7%)	0.001^∗∗^
	Incomplete improvement				12 (40.3%)	4 (13%)	
	No change				6 (20.0%)	18 (60%)	
	Worsened				0 (0.0%)	6(20%)	

Abbreviations: PND: postnasal drip; BLF: bronchial lavage fluid. ^∗^Data analyzed with McNemar in a significance level of *p* < 0.05. ^∗∗^Data analyzed with Wilcoxon in a significance level of *p* < 0.05. ^∗∗∗^Data analyzed with the Student's *t*-test in a significance level of *p* < 0.05. ^∗∗∗∗^Data analyzed with Wilcoxon in a significance level of *p* > 0.05.

## Data Availability

All data used in the research are available in any situation you need that can be accommodated as soon as requested.
